# Chemical Profiling and Discrimination of Essential Oils from Six *Ferula* Species Using GC Analyses Coupled with Chemometrics and Evaluation of Their Antioxidant and Enzyme Inhibitory Potential

**DOI:** 10.3390/antibiotics9080518

**Published:** 2020-08-14

**Authors:** Fadia S. Youssef, Munira A. Mamatkhanova, Nilufar Z. Mamadalieva, Gokhan Zengin, Salima F. Aripova, Elham Alshammari, Mohamed L. Ashour

**Affiliations:** 1 Department of Pharmacognosy, Faculty of Pharmacy, Ain Shams University, Cairo 11566, Egypt; fadiayoussef@pharma.asu.edu.eg; 2 Institute of the Chemistry of Plant Substances, Academy of Sciences of RUz, Mirzo Ulugbek str. 77, Tashkent 100170, Uzbekistan; munir_05@mail.ru (M.A.M.); nmamadalieva@yahoo.com (N.Z.M.); salima_aripova@mail.ru (S.F.A.); 3 Department of Bioorganic Chemistry, Leibniz Institute of Plant Biochemistry, Weinberg 3, D-06120 Halle (Saale), Germany; 4 Department of Biology, Science Faculty, Selcuk University, 42130 Konya, Turkey; gokhanzengin@selcuk.edu.tr; 5 Department of Pharmacy Practice, College of Pharmacy, Princess Nourah bint Abdulrahman University, Riyadh 11671, Saudi Arabia; ejalshammari@pnu.edu.sa

**Keywords:** *Ferula*, GC, essential oils, chemometrics, antioxidant activity, enzyme inhibition

## Abstract

The differences in the composition of essential oils obtained from the aerial parts of six *Ferula* species viz., *F. caratavica* (*Fc*), *F. kuchistanica* (*Fk*), *F. pseudoreoselinum* (*Fp*), *F. samarcandica* (*Fs*), *F. tenuisecta* (*Ft*) and *F. varia* (*Fv*) were detected both qualitatively and semi-quantitatively using GC-MS and GC-FID analyses. One hundred and six metabolites were identified that account for 92.1, 96.43, 87.43, 95.95, 92.90 and 89.48% of *Fc*, *Fk*, *Fp*, *Fs*, *Ft* and *Fv* whole essential oils, respectively. The data from the GC-MS analyses were subjected to unsupervised pattern recognition chemometric analysis utilizing principal component analysis (PCA) to improve the visualization of such differences among the six species. *Fk* and *Ft* are very closely related to each other and were gathered together in one cluster. The antioxidant potential was assessed in vitro using different assays including 2,2′-azino-bis (3-ethylbenzothiazoline-6-sulphonic acid) (ABTS), cupric reducing antioxidant capacity (CUPRAC), ferric reducing power (FRAP) and phosphomolybdenum (PM) assays. *Ft* and *Fp* exhibited the most notable antioxidant properties as evidenced by their pronounced activities in most of the antioxidant assays performed, followed by *Fc*. *Fk* showed the most effective tyrosinase inhibitory potential, which was estimated as 119.67 mgKAE/g oil, while *Fp* exhibited the most potent α-amylase inhibitory potential, which was equivalent to 2.61 mmol ACAE/g oil. Thus, it was concluded that *Ferula* species could serve as a promising natural antioxidant drug that could be included in different products and spices to alleviate hyperglycemia and used as a natural ingredient in pharmaceutical cosmetics to counteract hyperpigmentation.

## 1. Introduction

Essential oils comprise a mixture of secondary metabolites, which are biosynthesized by aromatic plants as natural protectants [1]. The role of essential oils is not restricted to protection as they also offer many therapeutic benefits to humans that can exceed the benefits provided by the dried herbs on their own [2]. Recently, they have become well known as a part of traditional medicine for the treatment of a plethora of human ailments, in aromatherapy, as well as in spices with high nutritive value [3]. In addition, many essential oils as well as plant extracts have shown significant antioxidant potential [4,5,6]. New sources of medicinal agents that are effective and safe as well as selective has recently become the main target in drug discovery. Medicinal plants in general, and their volatile constituents in particular, act as a very important sources for the production of a huge number of biologically active agents, which are attractive chemical leads that are promising therapeutic agents for the alleviation of many ailments [7,8]. Many biological activities have been ascribed to the volatile constituents obtained from a variety of plants such as antinociceptive, anticancer, antiphlogistic, antiviral, antioxidant, antimicrobial, antimycotic, antiparasitic and insecticidal activities [9]. Moreover, the volatile constituents of plants are highly popular in the food, cosmetic and pharmaceutical industries because of their broad acceptance by consumers, relative safety, and their potential multipurpose effect [10,11].

The Apiaceae family is well-known for its rich aromatic plants, which are categorized under approximately 112 genera and nearly 316 species. Anise, chervil, celery, coriander, cumin, caraway, dill, fennel, ferula and galabanum are significant members of this family and they are characterized by their notable odor owing to the presence of considerable amounts of essential oils or the oleoresin predominant in their different organs [3]. These plants are widely used for culinary purposes either for their aroma or as nutrients [12].

*Ferula* constitutes the third largest genus in the Apiaceae family with nearly 180 species. The members of this genus are very popular for their essential oils, which are recognized as having many biological activities including antibacterial, antifungal, antiviral, antispasmodic, anticonvulsant, and antioxidant activity as well as having high nutritive value [13,14].

This study aimed to investigate the contents of the essential oil from six *Ferula* species growing in Uzbekistan, namely, *F. caratavica* (*Fc*), *F. kuchistanica* (*Fk*), *F. pseudoreoselinum* (*Fp*), *F. samarcandica* (*Fs*), *F. tenuisecta* (*Ft*) and *F. varia* (*Fv*) using GC analyses. Discrimination of these species was carried by coupling the data obtained from GC-analyses with chemometrics employing unsupervised pattern recognition techniques represented by principal component analysis (PCA). Furthermore, the antioxidant potential of the different essential oil samples using different assays, namely, 2,2′-azino-bis (3-ethylbenzothiazoline-6-sulphonic acid) (ABTS), cupric reducing antioxidant capacity (CUPRAC), ferric reducing power (FRAP), and the phosphomolybdenum (PM) assay were evaluated *in vitro*. In addition, an evaluation of the possible enzymatic inhibitory activities of essential oils against tyrosinase and α-amylase was done using standard in vitro bioassays.

## 2. Results and Discussion

### 2.1. Qualitative and Semi-quantitative Determinations by GC-MS and GC-FID

**Figure 1:**
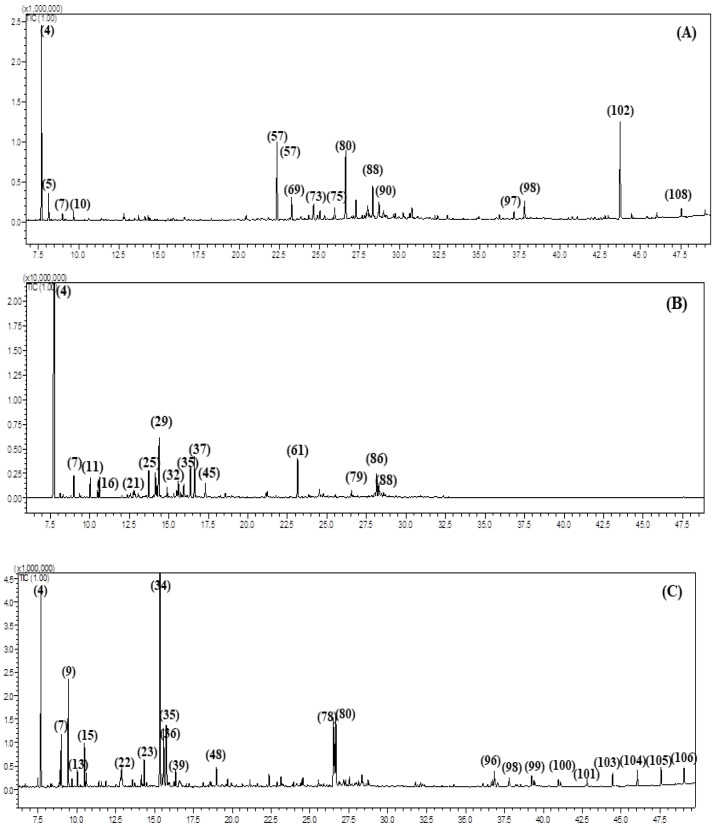
GC-MS chromatograms of *F. caratavica* (**A**), *F. kuchistanica* (**B**) and *F. pseudoreoselinum* (**C**).

**Figure 2:**
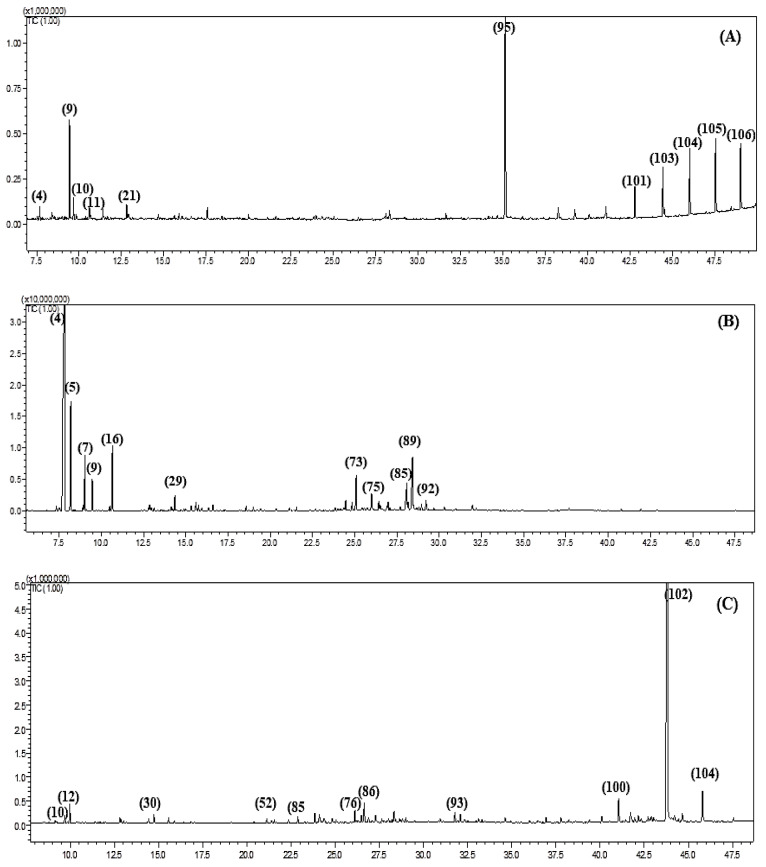
GC-MS chromatograms of *F. samarcandica* (**A**), *F. tenuisecta* (**B**) and *F. varia* (**C**).

**Figure 3:**
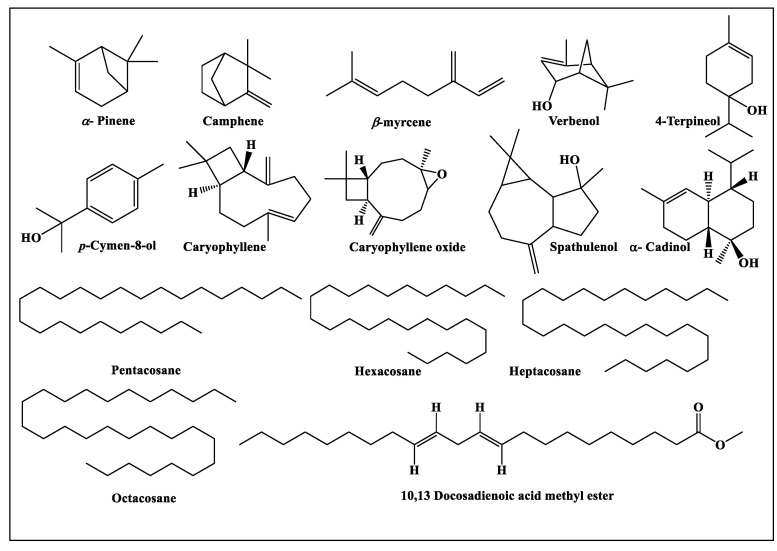
Main secondary metabolites in the *Ferula* species.

The differences in the composition of the essential oils obtained from the aerial parts of *Fc*, *Fk*, *Fp*, *Fs*, *Ft* and *Fv* were detected both qualitatively and quantitatively using GC-MS and GC-FID analyses, respectively. All of the essential oils are yellow in color and possess a characteristic odor. Characterization of the essential oils using GC analyses revealed the presence of 106 metabolites (Table 1, Figure 1 and Figure 2) that account for 92.10, 96.43, 87.43, 95.95, 92.90 and 89.48% of *Fc*, *Fk*, *Fp*, *Fs*, *Ft* and *Fv* whole essential oils, respectively. Twenty-nine compounds were detected in *Fc* with α-pinene (21.17%), 10,13 docosadienoic acid methyl ester (15.20%), β-caryophyllene oxide (13.23%) and caryophyllene (10.88%) representing the predominant compounds. Meanwhile, thirty-nine compounds were identified in *Fk* essential oil with α-pinene (36.79%) and verbenol (8.49%) being the major compounds. In *Fp*, forty-five compounds were characterized with 4-terpineol (16.28%), α-pinene (10.99%), β-myrcene (6.04%), β-caryophyllene oxide (5.69%), p-cymen-8-ol (5.36%) and spathulenol (5.34%) as the main metabolites in the oil. Furthermore, 15 compounds were determined in *Fs* oil with the main compounds, palmitic acid, β-myrecene, heptacosane, octacosane, hexacosane and pentcosane accounting for 39.09, 10.75, 10.27, 9.60, 8.99 and 6.29%, respectively. For *Ft*, 62 compounds were detected of which α-pinene (42.0%), camphene (8.34%) and α-cadinol (8.14%) exist in high percentages in the oil. Finally, 25 compounds were identified in the *Fv* oil with 10,13 docosadienoic acid methyl ester (69.61%) constituting the major component (Figure 3). From the data shown in Table 1, it was concluded that monoterpenes are the predominate class of essential oil metabolites in *Fc*, *Fk* and *Ft*, where they represents 24.90, 42.91 and 61.95%, respectively, while oxygenated monoterpenes are the dominant class of metabolites in *Fp* (35.60%), and they also exist in a high percentage in *Fk* (34.82%). On the contrary, fatty acids are highly predominate in *Fs* and *Fv* and account for 82.55 and 79.84%, respectively.

### 2.2. Chemometric Analysis

**Figure 4:**
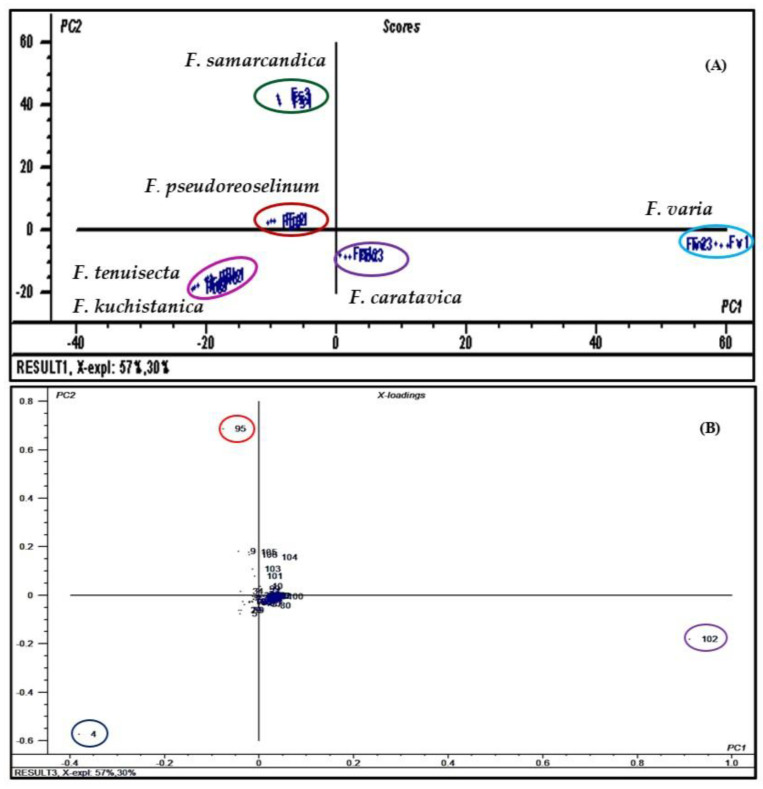
Score plot (**A**) and loading plot (**B**) of GC data obtained from *F. caratavica*, *F. kuchistanica*, *F. pseudoreoselinum*, *F. samarcandica*, *F. tenuisecta* and *F. varia* essential oil analyses using principal component analysis (PCA). In the loading plot, compounds are given numbers as in Table 1 where the major discriminatory signals are α-pinene **(4),** palmitic acid **(95)** and 10,13-docosadienoic acid methyl ester **(102)**.

It is extremely difficult to identify the qualitative and quantitative differences between the *Ferula* species under evaluation with the naked eye. So, the data obtained from GC analyses were subjected to unsupervised pattern recognition chemometric analysis utilizing PCA to improve the visualization of these differences. The results of the PCA, as represented by the obtained score plot shown in Figure 4A effectively discriminated the six *Ferula* species into five clusters along the first component (PC1) and the second component (PC2) that account for 57% and 30%, respectively, or 87% of the total variance. From the obtained results, it is obvious that both *Fk* and *Ft* are very closely related to each other as they are gathered together in one cluster in the lower left quadrant. However, PC1 successfully discriminated between *Fk* and *Ft* with negative values of PC1 as they are located in the lower left quadrant and *Fc* and *Fv*, which show positive values of PC1 are located in the lower right quadrant. Meanwhile, PC2 significantly discriminated between *Fk* and *Ft*, which show negative values of PC2 as they are located in the lower left quadrant and between *Fs* and *Fp*, which show positive values of PC2 and are located in the upper left quadrant. Furthermore, both PC1 and PC2 significantly discriminated between *Fc* and *Fv*, which show positive values for PC1 and negative values for PC2 as they are located in the lower right quadrant and between *Fs* and *Fp*, displaying negative values for PC1 and positive values for PC2 as they are located in the upper left quadrant. The major discriminatory signals are α-pinene, 10,13-docosadienoic acid methyl ester and palmitic acid as revealed in the loading plot shown in Figure 4B.

The Pearson correlation coefficient (r) between the essential oil contents of different studied samples indicated that *Fc* had a highly significant positive correlation with *Ft* (r = 0.71), *Fk* (r = 0.58), *Fv* (r = 0.47) and *Fp* (r = 0.35), while a non-significant negative correlation was observed between *Fc* and *Fs* (the highest correlations were observed between *Ft* and *Fk* (r = 0.89, *p* < 0.001), between *Fc* and *Ft* (r = 0.71, *p* < 0.001), and between *Fc* and *Fk* (r = 0.58, *p* < 0.001) as seen in Table 2. These data indicate that three samples, *Ft*, *Fk*, and Fc have highly similar essential oil content.

### 2.3. Biological Evaluation

#### 2.3.1. Antioxidant Potential of Different *Ferula* Species

The antioxidant potential of the different essential oil samples was performed in vitro using the 2,2′-azino-bis(3-ethylbenzothiazoline-6-sulfonic acid) (ABTS), the cupric ion reducing antioxidant capacity (CUPRAC), The ferric reducing antioxidant power (FRAP) and the phosphomolybdenum method (PM) assays. The results displayed in Table 3 reveal that most of the samples showed considerable antioxidant potential in the performed assays. *Fc* (41.36 mgTE/g oil) exhibited the most antioxidant activity in ABTS assays, followed by *Fk* (29.12 mgTE/g oil) and *Ft* (28.03 mgTE/g oil). However, in CUPRAC assay, *Fp* (289.45 mgTE/g oil) showed the most superior antioxidant potential followed by *Ft* (278.87 mgTE/g oil) and *Fk* (120.43 mgTE/g oil). Furthermore, *Ft* exhibited the most significant antioxidant power in both FRAP and PM assays with antioxidant activity equivalent to 136.81 mgTE/g oil and 78.66 mmolTE/g oil, respectively, followed by *Fp*, which showed antioxidant potential of 121.64 mgTE/g oil and 50.86 mmolTE/g oil in FRAP and PM assays, respectively. Thus, it can be concluded that the essential oil from both *Ft* and *Fp* exhibited the most notable antioxidant properties as evidenced by their pronounced activities in most of the performed antioxidant assays, followed by *Fc*. α-Pinene, the predominant compound in *Ft* and *Fp* has previously been shown to possess notable antioxidant activity [15]. Additionally, the significant antioxidant activity found in this study, which can be interpreted as a result of the synergistic action between the different components that exist in the oils, was in accordance with that previously reported for many other *Ferula* species such as *F. microcolea*, *F. orantalis* and *F. communis.* Various mechanisms can be used to interpret antioxidant potential including the prohibition of chain initiation, peroxide decomposition, obstruction of continual hydrogen removal as well as the scavenging of free radical and uniting transition metal ion catalysts [3,16,17]. Additionally, α-pinene, the main constituent in both *Ft* and *Fp*, has previously been shown to be a potent antioxidant in both DPPH and FRAP assays, displaying EC_50_ values equal to 310 and 238 μg/mL, respectively [18].

#### 2.3.2. Tyrosinase and α-Amylase Inhibitory Potential

Tyrosinase enzyme is an oxidase enzyme containing copper that assists in the completion of the first two steps of mammalian melanogenesis, which leads to undesirable hyperpigmentation. Thus, the search for effective tyrosinase inhibitors has recently become vital so that they can be incorporated in cosmetics for effective skin whitening and to counteract hyperpigmentation [19]. *Fk* showed the most effective tyrosinase inhibitory potential, which was estimated as 119.67 mgKAE/g oil followed by *Fv*, which showed an inhibitory potential equivalent to 118.42 mgKAE/g oil, where KAE is a Kojic acid equivalent, a potent tyrosinase inhibitory drug. *Fv* oil is rich in 10,13 docosadienoic acid methyl ester, a polyunsaturated fatty acid, which greatly accounts for its promise as a tyrosinase inhibitor [20]. The underlying tyrosinase inhibitory mechanism mainly relies on the essential oils being rich in components that possess a hydrophobic portion that competitively inhibits the active sites of tyrosinase enzyme with subsequent interference of melanin synthesis. This inhibition may be achieved via interaction with Cu^+2^ that exists in the active sites of tyrosinase in addition to the prohibition of tautomerization to dopachrome triggered by the oil, which behaves as a reducing agent and blocks of the oxidation reaction during the formation of melanin intermediates during the conversion of tyrosinase/DOPA into melanin, thus reducing skin pigmentation [21].

**Figure 5:**
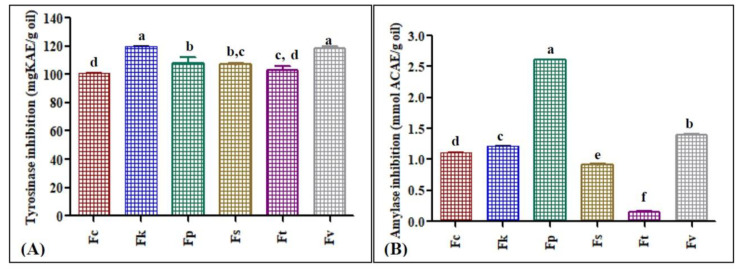
In vitro tyrosinase inhibition (**A**) and α-amylase inhibition (**B**) of the essential oil of different *Ferula* species, *F. caratavica* (*Fc*), *F. kuchistanica* (*Fk*), *F. pseudoreoselinum* (*Fp*), *F. samarcandica* (*Fs*), *F. tenuisecta* (*Ft*) and *F. varia* (*Fv*). Different letters (a–f) indicate significant differences in the tested *Ferula* species (*p* < 0.05).

The α-amylase enzyme is critical in assisting in the catalysis of the first steps in the conversion of starch into maltose, and subsequently to glucose [22,23]. Nowadays, α-amylase inhibitors are used in therapeutic approaches to counteract hyperglycemia. *Fp* and *Fv* exhibited the most potent α-amylase inhibitory potential as evidenced by their pronounced inhibitory activity, which was equivalent to 2.61 and 1.40 mmol ACAE/g oil, respectively, in which ACAE is the acarbose equivalent, a potent α-amylase inhibitor (Figure 5). 4-Terpineol as well as α-pinene, which predominate the essential oil of *Fp*, were previously reported to possess considerable α-amylase inhibitory activity [24]. Similarly, the potent α-amylase inhibitory potential is mainly due to the synergistic action between the different components, which is in accordance to different previously reported studies that confirmed the α-amylase inhibitory effect of different terpenes and different *Ferula* species such as *F. gummosa* essential oil [24,25].

## 3. Materials and Methods

### 3.1. Plant Material

Aerial parts (flowers, leaves and stems) of *F. caratavica* Regel & Schmalh (N2004), *F. pseudoreoselinum* (Regel & Schmalh.) Koso-Pol., p.p. (N1489), *F. tenuisecta* Korovin (N1488) were collected from the Tashkent region of Uzbekistan. *F. varia* (Schrenk ex Fisch., C.A.Mey. & Avé-Lall.) Trautv. (N1407) was collected from the Bukhara region (Uzbekistan), while *F. kuchistanica* Korovin (N1425) and *F. samarcandica* Korovin (N1919) were collected from the Samarkand region of Uzbekistan. The plants were collected during the flowering stage in June–July 2018. Their taxonomic authentication was accomplished by Dr. A. Nigmatullaev at the Institute of the Chemistry of Plant Substances (Tashkent, Uzbekistan).

### 3.2. Preparation of Essential Oil Samples

All the plant materials were air-dried in the shade for 7 days at room temperature and powdered using a mortar and pestle to get particles of a uniform, reduced size. Preparation of the essential oil samples was achieved by hydrodistillation of the air-dried aerial parts of the different *Ferula* species, *F. caratavica* (*Fc)*, *F. kuchistanica (Fk)*, *F. pseudoreoselinum* (*Fp)*, *F. samarcandica* (*Fs)*, *F.* tenuisecta *(Ft)* and *F. varia* (*Fv)* for 2 h by Clevenger-type apparatus. Anhydrous Na_2_SO_4_ was used to dehydrate the prepared essential oils, yielding 0.4, 0.7, 0.3, 0.3, 0.8 and 0.5 % v/w of dry weight for *Fc*, *Fk*, *Fp*, *Fs*, *Ft* and *Fv*, respectively. Then the various oil samples were maintained at −30 °C in dark-colored stoppered glasses until their analyses [26,27].

### 3.3. GC-FID and GC-MS Analyses

A Shimadzu GC-17A gas chromatograph (Shimadzu Corporation, Kyoto, Japan) with an FID detector and DB-5 fused-bonded cap column (Phenomenex; 29 m × 0.25 mm i.d., film thickness 0.25 µm; Torrance, California, USA) was utilized for the semi-quantitative determination of the different components of the essential oils using the normalization method to get the relative percentage of each component and applying GC-FID data that is highly sensitive using GC solution^®^ software ver. 2.4 (Shimadzu Corporation, Kyoto, Japan). The areas under the peaks (AUP) were determined using three independent runs where the total area is considered as 100%. Meanwhile, the Shimadzu GC-2010 plus gas chromatograph (Shimadzu Corporation, Kyoto, Japan) supplied with Rtx-5MS (Restek, Bellefonte, PA, USA) in addition to a quadrupole mass spectrometer was used for the identification of the essential oil different metabolites. Instrument settings were adjusted according to what was previously reported [28,29]. The Wiley Registry of Mass Spectral Data 8th edition, NIST MassSpectral Library (December 2011), and previously reported data were employed to confirm the identity of the compounds and the retention indexes were calculated to corroborate the identification of the volatile compounds [30,31].

### 3.4. Chemometric and ANOVA Analysis

To examine the differences between the essential oils’ components prepared from different *Ferula* species, the data collected from the different GC-MS spectra were subjected to chemometric analysis of unsupervised pattern recognition represented by PCA, which was processed by employing Unscrambler 9.7 (CAMO SA, Oslo, Norway) [28,32]. Meanwhile, other statistical analyses used for biological assessment were performed using ANOVA assay (with Tukey’s test, significant value: *p* < 0.05) and Xlstat 2017 software.

### 3.5. Biological Evaluation

#### 3.5.1. Determination of the Antioxidant Potential

The antioxidant activity of the different essential oil samples from different *Ferula* species was evaluated using ABTS, CUPRAC, FRAP and PM assays. These assays were performed following the methods described by Mamadalieva et al. [33]. The antioxidant activities were reported as Trolox equivalents and the samples were analyzed in triplicate.

#### 3.5.2. Determination of Enzyme Inhibitory Effects

The possible inhibitory potential of the essential oil samples was investigated against tyrosinase and α-amylase enzymes using standard in vitro bioassays as previously reported by Mamadalieva et al. [33] in which all the samples were analyzed in triplicate. Results are expressed in mgKAE/g oil for tyrosinase inhibitory activity and in mmol ACAE/g oil for α-amylase inhibition.

## 4. Conclusions

The essential oils obtained from different *Ferula* species, *F. caratavica*, *F. kuchistanica*, *F. pseudoreoselinum*, *F. samarcandica*, *F. tenuisecta* and *F. varia* showed significant variation as revealed by GC analyses. Furthermore, this variation became more clearly observable when coupled with a chemometric approach as represented by PCA used as an unsupervised pattern recognition technique. Additionally, the obtained essential oils showed notable antioxidant as well as tyrosinase and α-amylase inhibitory activities with variable degrees, which is mainly related to the differences in the secondary metabolites that predominate in the oils. Thus, it was concluded that the different *Ferula* species could serve as a promising natural antioxidant drug that could be included in different products and used as spices to alleviate hyperglycemia and as a natural ingredient in pharmaceutical cosmetics to counteract hyperpigmentation. Chemometric study based on gathering the different biological activities of many additional *Ferula* species will be considered. It is recommended that further in vivo studies such as animal and bioavailability studies be carried out to confirm the obtained results.

## Figures and Tables

**Table 1:** Composition of volatile oil in the aerial parts of *F. caratavica* (*Fc*), *F. kuchistanica* (*Fk*), *F. pseudoreoselinum* (*Fp*), *F. samarcandica* (*Fs*), *F. tenuisecta* (*Ft*) and *F. varia* (*Fv*).

Compound		RI		Content, (%)						Identification Methods
		Cal.	Rep.	*Fc*	*Fk*	*Fp*	*Fs*	*Ft*	*Fv*	
**1.**	n-Nonane	889	900	0.37	-	0.16	-	-	-	MS, RI,
**2.**	Tricyclene	913	913	-	-	-	-	0.37	-	MS, RI,
**3.**	3-Thujene	919	919	-	-	0.50	-	0.36	-	MS, RI, AU
**4.**	α-Pinene	925	925	21.17	36.79	10.99	1.5	42.0	-	MS, RI, AU
**5.**	Camphene	941	941	2.91	0.47	-	-	8.34	-	MS, RI,
**6.**	Sabinene	970	970	-	-	0.94	-	0.35	-	MS, RI,
**7.**	β-Pinene	973	973	0.82	1.88	3.11	-	3.59	-	MS, RI, AU
**8.**	6-Methyl-5-heptene-2-one	986	986	-	0.35	-	-	-	-	MS, RI, AU
**9.**	β-Myrcene	989	989	-	0.19	6.04	10.75	1.90	-	MS, RI,
**10.**	n-Decane	998	1000	1.00	0.16	0.47	2.44	0.06	0.62	MS, RI,
**11.**	α-Phellandrene	1003	1003	-	1.81	tr	-	-	-	MS, RI, AU
**12.**	(3E)-3-Hexenyl acetate	1007	1006	-	-	-	-	-	1.40	MS, RI,
**13.**	3-Carene	1009	1009	-	tr	0.97	-	0.07	-	MS, RI,
**14.**	2-Carene	1016	1018	-	-	tr	-		-	MS, RI,
**15.**	β-Cymene	1024	1025	-	tr	2.59	-	0.39	-	MS, RI,
**16.**	Limonene	1028	1028	-	1.77	0.83	1.15	4.80	-	MS, RI, AU
**17.**	τ-Terpinene	1059	1059	-	-	0.30	-	-	-	MS, RI, AU
**18.**	Linalool oxide	1074	1074	-	0.25	-	-	-	-	MS, RI,
**19.**	Terpinolene	1089	1089	-	-	-	-	tr	-	MS, RI,
**20.**	Dehydro-p-cymene	1090	1090	-	-	-	-	0.15	-	MS, RI,
**21.**	n-Undecane	1098	1100	tr	1.60	-	1.56	-	0.43	MS, RI,
**22.**	β-Linalool	1100	1100	tr	tr	2.03	-	0.54	-	MS, RI, AU
**23.**	*cis-p*-Menth-2,8-dienol	1108	1104	-	0.80	1.78	-	0.40	-	MS, RI,
**24.**	Fenchol	1116	1117	-	-	-	-	0.04	-	MS, RI,
**25.**	6-Camphenol	1128	1131	-	2.75		-	0.05	-	MS, RI,
**26.**	Limonene oxide	1135	1133	-	-	-	-	tr	-	MS, RI,
**27.**	4-Isopropenyl-1-methyl-2-cyclohexen-1-ol	1137	1142	-	0.32	0.49	-	0.12	-	MS, RI,
**28.**	L-pinocarveol	1141	1141	-	3.90	0.74	-	0.33	-	MS, RI,
**29.**	Verbenol	1148	1148	-	8.49	-	-	1.25	-	MS, RI,
**30.**	*trans*-2-Nonenal	1160	1161	-	-	-	-	0.03	0.73	MS, RI,
**31.**	3-Pinanone	1163	1160	-	tr	-	-	-	-	MS, RI,
**32.**	Verbenone	1165	1173	-	1.43	-	-	-	-	MS, RI,
**33.**	Borneol	1169	1169	-	-	-	-	0.11	-	MS, RI,
**34.**	4-Terpineol	1179	1179	-	0.39	16.28	-	0.36	-	MS, RI,
**35.**	p-Cymen-8-ol	1186	1186	tr	2.85	5.36	-	0.80	0.49	MS, RI,
**36.**	α-Terpineol	1193	1193	-	0.52	5.00	-	0.41	-	MS, RI,
**37.**	Myrtenol	1199	1199	-	2.30	0.65	-	0.21	-	MS, RI,
**38.**	*cis*-Geraniol	1210	1210	-	-	0.35	-	tr	-	MS, RI,
**39.**	Verbenone	1214	1214	-	3.95	1.11	-	0.18	-	MS, RI,
**40.**	Fenchyl acetate	1222	1223	-	4.51	-	-	-	-	MS, RI,
**41.**	*cis*-Carveol	1225	1220	-	tr	-	-	0.46	-	MS, RI,
**42.**	β-Citronellol	1230	1230	-	-	-	-	0.05	-	MS, RI,
**43.**	*trans*-Carveol	1234	1229	-	-	-	-	0.04	-	MS, RI,
**44.**	Thymol methyl ether	1237	1237	-	0.19	0.23	-	0.03	-	MS, RI,
**45.**	D-Carvone	1249	1249	-	1.68	-	-	tr	-	MS, RI,
**46.**	Nerol	1252	1251	-	tr	-	-	tr	-	MS, RI,
**47.**	Bornyl acetate	1290	1290	-	0.49	0.39	-	0.31	-	MS, RI,
**48.**	(-)-trans-Pinocarvyl acetate	1305	297	-	-	1.19	-	-	-	MS, RI,
**49.**	Carvacrol	1306	1306	-	tr	-	-	-	-	MS, RI,
**50.**	α-Cubebene	1353	1353	-	-	-	-	0.16	-	MS, RI,
**51.**	D-longifolene	1370	1370	-	tr	-	-	-	-	MS, RI,
**52.**	α-Copaene	1380	1380	-	0.38	0.41	-	0.18	0.39	MS, RI,
**53.**	β-Gurjunene	1386	1388	-	4.81	-	-	-	-	MS, RI,
**54.**	β-Bourbonene	1390	1390	-	-	-	-	-	tr	MS, RI,
**55.**	β-Elemene	1395	1395	-	-	-	-	0.24	tr	MS, RI,
**56.**	Jasmone	1403	1399	-	tr	-	-	-	-	MS, RI,
**57.**	β-Caryophyllene	1425	1425	10.88	-	0.91	-	0.08	tr	MS, RI, AU
**58.**	τ-Elemene	1438	1438	-	-	-	-	0.12	-	MS, RI,
**59.**	Patchoulene	1440	1440	tr	-	0.38	-		-	MS, RI,
**60.**	Alloaromadendrene	1447	1442	-	-	-	-	-	0.63	MS, RI,
**61.**	Geranyl acetone	1456	1455	-	4.48	0.58	-	-	-	MS, RI,
**62.**	α-Humulene	1461	1461	2.98	tr	-	-	0.04	-	MS, RI,
**63.**	τ-Muurolene	1467	1467	-	0.27	-	-	0.21	0.87	MS, RI,
**64.**	α-Curcumene	1487	1486	-	tr	-	-		-	MS, RI,
**65.**	Germacrene D	1489	1489	-	-	-	-	0.13	-	MS, RI,
**66.**	β-Eudesmene	1495	1495	-	-	-	-	0.20	-	MS, RI,
**67.**	β-Guaiene	1503	1500	0.65	-	tr	-	0.38	tr	MS, RI,
**68.**	α-Muurolene	1508	1508	-	-	0.35	-	0.74	-	MS, RI,
**69.**	Cuparene	1514	1513	3.09	0.23	-	-	-	-	MS, RI,
**70.**	α-Selinene	1514	1517	-	tr	-	-	0.07	0.34	MS, RI,
**71.**	τ-Cadinene	1523	1521	-	-	-	-	0.75	0.50	MS, RI,
**72.**	β-Cadinene	1524	1529	tr	-	-	-	-	-	MS, RI,
**73.**	δ-Cadinene	1531	1531	1.37	-	-	tr	3.07	-	MS, RI,
**74.**	Elemol	1557	1577	-	-	-	-	tr	-	MS, RI,
**75.**	Nerolidol	1566	1564	1.70	-	-	-	1.74	-	MS, RI,
**76.**	Germacrene B	1572	1569	-	-	-	-	-	1.07	MS, RI,
**77.**	Germacrene D-4-ol	1585	1583	-	-	-	-	0.75	-	MS, RI,
**78.**	Spathulenol	1587	1587	-	-	5.34	-	0.38	0.65	MS, RI,
**79.**	Globulol	1590	1590	-	1.06	3.25	-	0.18	-	MS, RI,
**80.**	Caryophyllene oxide	1594	1594	13.23	tr	5.69	-		2.14	MS, RI, AU
**81.**	Guaiol	1602	1602	-	-	-	-	-	0.53	MS, RI,
**82.**	Cubenol	1606	1605	-	tr	-	-	1.13	-	MS, RI,
**83.**	β-Eudesmol	1612	1613	-	-	-	-	0.20	-	MS, RI,
**84.**	τ-Eudesmol	1631	1631	-	-	0.66	-		-	MS, RI,
**85.**	τ-Muurolol	1652	1652	2.03	-	-	tr	3.49	1.02	MS, RI,
**86.**	δ-Cadinol	1656	1656	tr	2.82	-	tr	0.79	-	MS, RI,
**87.**	τ-Muurolol	1665	1661	4.64	-	-	-		-	MS, RI,
**88.**	α-Eudesmol	1666	1662	-	2.54	-	-		1.01	MS, RI,
**89.**	α-Cadinol	1669	1669	-	-	-	-	8.14	-	MS, RI,
**90.**	Cedr-8-en-13-ol	1682	1688	2.17	-	-	-		-	MS, RI,
**91.**	α-Bisabolol	1692	1692	-	-	-	-	0.56	-	MS, RI,
**92.**	Farnesol	1726	1725	0.82	-	-	-	1.13	-	MS, RI,
**93.**	Hexadecanal	1817	1819	-	-	-	-	-	1.16	MS, RI,
**94.**	Hexahydrofarnesyl acetone	1845	1845	-	tr	-	-	-	-	MS, RI,
**95.**	Palmitic acid	1977	1975	-	-	-	39.03	-	-	MS, RI, AU
**96.**	trans-9-Octadecen-1-ol	2068	2068	-	-	1.31	-	-	-	MS, RI,
**97.**	Heptadecanoic acid ethyl ester	2082	2082	1.06	-	-	-	-	-	MS, RI,
**98.**	*trans*-Phytol	2120	2122	2.69	-	0.42	-	-	-	MS, RI,
**99.**	Docosane	2200	2200	-	-	0.60	1.47	-	-	MS, RI,
**100.**	Tricosane	2301	2300	-	-	0.34	tr	-	2.66	MS, RI,
**101.**	Tetracosane	2395	2400	-	-	0.51	4.49	-	-	MS, RI,
**102.**	10,13 Docosadienoic acid methyl ester	2449	2449	15.2	-	-	-	-	69.61	MS, RI,
**103.**	Pentacosane	2498	2500	0.66	-	0.95	6.26	-	-	MS, RI,
**104.**	Hexacosane	2598	2600	0.63	-	1.10	8.99	-	3.23	MS, RI,
**105.**	Heptacosane	2697	2700	1.26	-	1.35	10.27	-	-	MS, RI,
**106.**	Octacosane	2790	2800	0.77	-	1.13	9.60	-	tr	MS, RI,
**Monoterpene hydrocarbons**				24.9	42.91	26.27	13.40	61.95	-	
**Oxygenated monoterpene**				tr	34.82	35.60	-	5.69	0.49	
**Sesquiterpene hydrocarbons**				18.97	5.69	2.05	tr	6.37	3.80	
**Oxygenated sesquiterpene**				24.59	10.9	15.52	tr	18.49	5.35	
**Others**				23.64	2.11	7.87	82.55	0.40	79.84	
**Total**				92.10	96.43	87.31	95.95	92.90	89.48	

Compounds were identified based on a comparison of the compounds’ mass spectral data and retention indices with those of the NIST Mass Spectral Library (December 2011), the Wiley Registry of Mass Spectral Data, 8th edition and by comparison with the authentic standard (AU). The content (%) was calculated using the normalization method based on the GC-FID data generated from the average of three independent chromatographic runs.

**Table 2:** The Pearson correlation matrix of the essential oils content of different samples.

	*Fc*	*Fk*	*Fp*	*Fs*	*Ft*	*Fv*
***Fc***	-	0.58 ***	0.35 ***	−0.02	0.71 ***	0.47 ***
***Fk***	0.58 ***	-	0.43 ***	−0.02	0.89 ***	−0.03
***Fp***	0.35 ***	0.43 ***	-	0.05	0.45 ***	−0.03
***Fs***	−0.02	−0.02	0.05	-	−0.002	−0.02
***Ft***	0.71 ***	0.89 ***	0.45 ***	−0.002	-	−0.03
***Fv***	0.47 ***	−0.03	−0.03	−0.02	−0.03	-

The data is represented as the r value of the correlation coefficient and *** is the level of significance, *p* < 0.001.

**Table 3:** Antioxidant activities of the essential oil samples of *Ferula* species using the 2,2′-azino-bis(3-ethylbenzothiazoline-6-sulfonic acid) (ABTS), the cupric ion reducing antioxidant capacity (CUPRAC), The ferric reducing antioxidant power (FRAP) and the phosphomolybdenum method (PM) assays.

Samples	ABTS (mgTE/g Oil)	CUPRAC (mgTE/g Oil)	FRAP (mgTE/g Oil)	PM (mmolTE/g Oil)
*F. caratavica* (Fc)	41.36 ± 1.27 ^a^	83.54 ± 3.13 ^c^	47.34 ± 0.65 ^e^	5.59 ± 0.01 ^f^
*F. kuchistanica* (Fk)	29.12 ± 0.85 ^b^	120.43 ± 9.36 ^b^	80.74 ± 0.25 ^c^	36.42 ± 0.07 ^c^
*F. pseudoreoselinum* (Fp)	22.68 ± 1.03 ^c^	289.45 ± 7.30 ^a^	121.64 ± 0.01 ^b^	50.86 ± 0.07 ^b^
*F. samarcandica* (Fs)	11.84 ± 1.37 ^d^	74.39 ± 4.73 ^c,d^	43.21 ± 0.48 ^f^	14.37 ± 0.04 ^e^
*F. tenuisecta* (Ft)	28.03 ± 3.89 ^b^	278.87 ± 8.51 ^a^	136.81 ± 1.98 ^a^	78.66 ± 0.15 ^a^
*F. varia* (Fv)	7.04 ± 0.47 ^e^	65.90 ± 1.66 ^d^	55.00 ± 0.18 ^d^	15.33 ± 0.07 ^d^

Values are reported as mean ± S.D of three parallel measurements. TE: Trolox equivalents. Different superscripts (a–f) indicate significant differences in the tested *Ferula* species (*p* < 0.05).
